# Pancreatic cancer extracellular vesicles stimulate Schwann cell activation and perineural invasion in vitro via IL-8/CCL2

**DOI:** 10.1007/s44164-025-00083-w

**Published:** 2025-03-07

**Authors:** Emory Gregory, Isabel Powers, Azemat Jamshidi-Parsian, Robert J. Griffin, Younghye Song

**Affiliations:** 1 Department of Biomedical Engineering, University of Arkansas, Fayetteville, AR USA; 2 Department of Radiation Oncology, University of Arkansas for Medical Sciences, Little Rock, AR USA

**Keywords:** Pancreatic cancer, Perineural invasion, Tissue engineering, Glia, Extracellular vesicles

## Abstract

**Purpose:**

Pancreatic ductal adenocarcinoma (PDAC) remains a leading cause of cancer-related deaths, and perineural invasion (PNI), in which cancer cells infiltrate nerves, enables metastasis in most patients. PNI is largely attributed to Schwann cells (SC) that, when activated, accelerate cancer cell migration towards nerves. However, this cancer-associated reprogramming is generally under-appreciated. Additionally, tumor extracellular vesicle (EV) facilitation of cancer aggravation is well documented, but more investigation is required to better understand their role in PNI. Here, we assessed whether PDAC EVs mediate PNI via SC activation using tissue-engineered in vitro platforms and PANC-1 and HPNE human cell lines as models.

**Methods:**

NanoSight, Luminex®, and proteomic-pathway analyses characterized tumor (PANC-1) and healthy cell (HPNE) EVs. Human Schwann-like cells (sNF96.2) were embedded in decellularized nerve matrix hydrogels and then treated with EVs and a cargo-function-blocking antibody. Immunofluorescence and Luminex® multiplex assays assessed Schwann cell activation. Subsequently, sNF96.2 cells were co-cultured with EVs and either PANC-1 or HPNE cells; Transwell® invasion assays with SC-conditioned media were also conducted to establish a mechanism of in vitro PNI.

**Results:**

PANC-1 EVs contained higher levels of interleukin-8 (IL-8) signaling-associated proteins than HPNE EVs. Within nerve-mimetic in vitro testbeds, PANC-1 EVs promoted sNF96.2 activation per cytoskeletal marker alterations and secretion of pro-tumorigenic cytokines, e.g., chemokine ligand-2 (CCL2), via IL-8 cargoes. Furthermore, the IL-8/CCL2 axis heightened PANC-1 invasiveness.

**Conclusion:**

These findings highlight the potential role of PDAC EVs in PNI, which necessitates continued preclinical assessments with increased biodiversity to determine the efficacy of targeting IL-8/CCL2 for PNI.

**Supplementary Information:**

The online version contains supplementary material available at 10.1007/s44164-025-00083-w.

## Introduction

The incidence of pancreatic ductal adenocarcinoma (PDAC) in the USA has continued to rise by 1% each year since 2000 and carries with it a 10% 5-year survival rate across stages [1]. Within the last two decades, researchers have revealed perineural invasion (PNI), in which cancer cells penetrate adjacent nerves, as a key factor in the devastation of 70–95% of PDAC cases [1]. While physician-scientists continue to innovate novel diagnosis methods, there is a dire need for effective therapeutics for patients experiencing PNI [2].

Cells within the tumor microenvironment regulate cancer cell invasiveness; for instance, Schwann cells (SC) predominately drive PNI initiation and progression in PDAC [3]. These glial cells regularly maintain peripheral nerve health, but under distress, they dedifferentiate into an activated phenotype, altering their presentation of activation marker glial fibrillary acidic protein (GFAP). Activated SCs modify their secretome, e.g., chemokine ligand-2 (CCL2), matrix metalloproteinase-2 (MMP-2), interleukin (IL-)8, IL-6, IL-1β, and glial-derived neurotrophic factor (GDNF), to propagate repair; however, cancer cells may exploit these signals to enhance PNI [4, 5]. While it is clear SCs instigate PNI, the mode by which SCs are activated by tumors remains unclear.

Meanwhile, extracellular vesicles (EV) produced by tumor cells (TEV), including exosomes (diameters 30–150 nm) and microvesicles (diameters 100–1000 nm), modulate microenvironmental reprogramming to enhance tumor metastatic potential [6]. In PDAC, TEVs enhance cancer cell migration, while colon cancer exosomes activate SCs, increasing cancer cell proliferation in vivo [7, 8]. Blocking exosome biogenesis even appears to limit cervical and head and neck tumor innervation, a common comorbidity to PNI in which neurites infiltrate solid tumors [9, 10]. Still, more detailed investigation is required to validate the role of TEVs in PDAC PNI.

Previously, most knowledge of cancer-nerve crosstalk was derived from clinical observations, while the mechanisms behind PNI have largely been analyzed with in vivo assessments [11]. Unfortunately, in vivo models often produce simultaneous instances of PNI and tumor innervation, making it difficult to dissect mechanisms of each and identify therapeutic strategies. Moreover, animal studies are expensive, tend to have lower reproducibility, and struggle to translate to human anatomy and physiology [12, 13]. Researchers have recently shifted their focus to engineered in vitro models that provide highly reproducible outcomes and modulate the tumor microenvironment in a controlled manner [14]. For example, hydrogel platforms offer a three-dimensional environment for physiological cell behavior and have been utilized in studying cancer-nerve crosstalk [15, 16]. Decellularized extracellular matrix (dECM) hydrogels, which are derived tissues stripped of native cells through physical, chemical, or enzymatic processes, replicate the biochemical composition and structural architecture of the tumor microenvironment particularly well, yet few studies have utilized these unique testbeds to investigate PNI [17]. To bridge this knowledge gap, we previously established a dECM peripheral nerve hydrogel for studying neuropathology, emphasizing its value in modeling PNI [18].

In the current study, we implemented our optimized dECM nerve in vitro culture platform to examine the role of pancreatic TEVs in activating SCs to mediate PNI [18]. Using PANC-1 as a model PDAC cell type, we found IL-8 to be significantly elevated in TEVs (PANC-1) over healthy cell (HPNE) EVs and regulated SC (sNF96.2) activation, in which state SCs heavily upregulate the release of IL-8 and CCL2. Through this IL-8/CCL2 cascade, TEVs, but not EVs, primed SCs to significantly enhance PDAC cell invasiveness. This work showcases proof of a potential mechanism by which TEVs promote PDAC PNI.

## Materials and methods

### Cells

Human sNF96.2 Schwann-like, hTERT-HPNE pancreatic duct epithelial, and PANC-1 pancreatic ductal carcinoma cells were purchased from ATCC (Manassas, VA). STR authentication was performed by ATCC, and experiments were performed with mycoplasma-free cells determined using MycoAlert Plus (Lonza LT07-705 and LT07-518). HPNE cells were cultured in 75% glucose-free Dulbecco’s Modified Eagle Medium (DMEM; Thermo 11,966,025) and 25% M3 base (Incell M300) supplemented with 5% fetal bovine serum (FBS; Atlanta Biologicals S11150), 10 ng/ml human recombinant epidermal growth factor (Fisher PHG0311), 5 mM glucose (Fisher A2494001), and 750 ng/ml puromycin (Fisher A1113803). PANC-1 cells were maintained in DMEM (Thermo 11,965,092) with 10% FBS, 1% penicillin–streptomycin (ATCC PCS999002), and 4.5 g/L glucose. SCs were cultured in DMEM with 10% FBS, 1% penicillin–streptomycin, 2 µM forskolin (Sigma-Aldrich F6886), 20 ng/ml neuregulin (Millipore 492028), and 1 g/L glucose. All cell lines were maintained in culture at 37 °C and 5% CO_2_ and sub-cultured using trypsin–EDTA.

### EV isolation and lysis

**Fig. 1:**
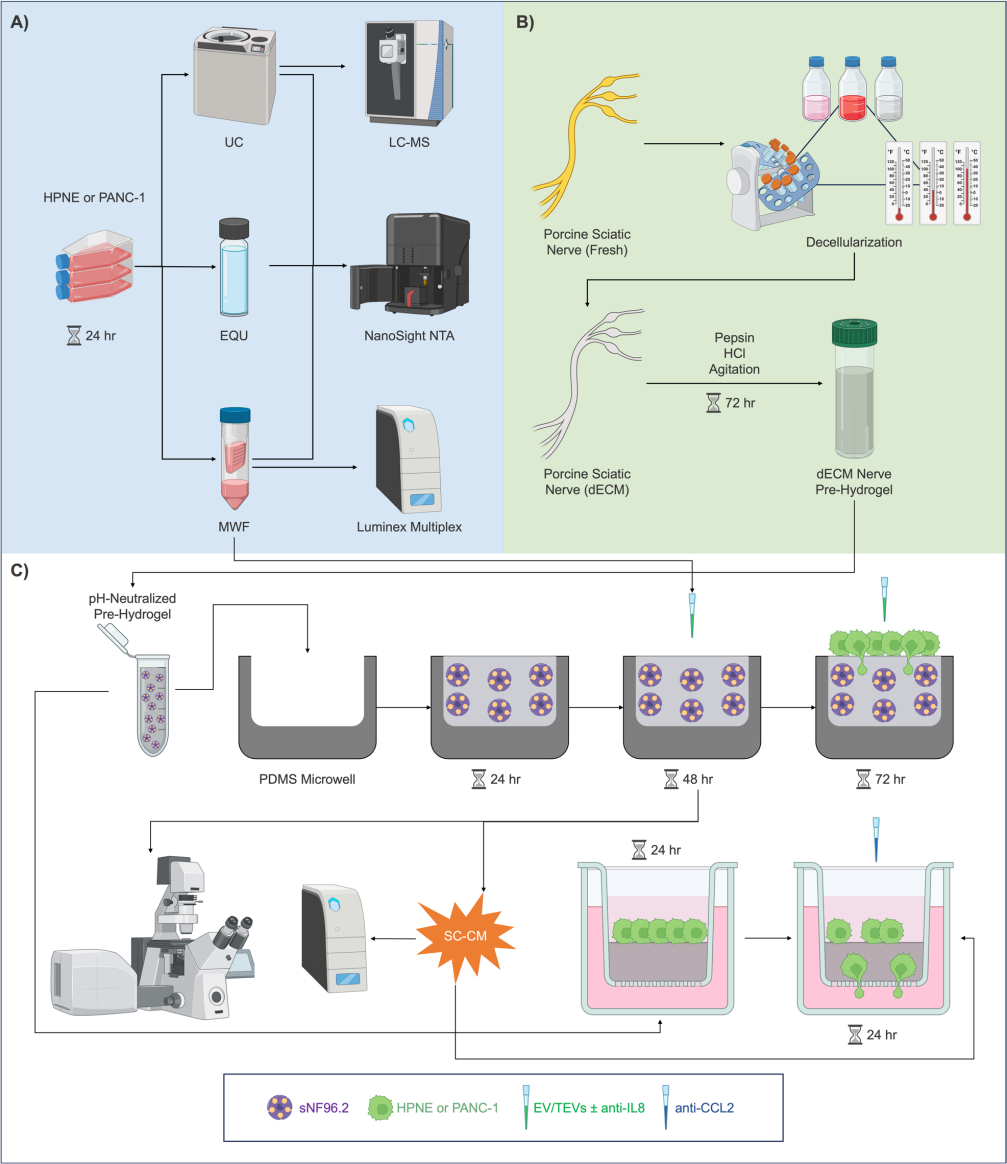
Experimental design. **A** HPNE or PANC-1 was incubated with serum-free media, which after 24 h were collected and allocated to either UC, EQU, or MWF isolation of EVs. All processed samples were analyzed using NanoSight Nanoparticle Tracking, and proteomics was run with UC samples. Following initial analysis, MWF samples were probed via Luminex multiplex and reserved for future in vitro assays. **B** Porcine sciatic nerves were subjected to various chemical and enzymatic with and without agitation at ranging temperatures in order to decellularize the tissues. Resulting nerves were digested into nerve dECM pre-hydrogel solutions for in vitro experiments. **C** sNF96.2 SCs (purple) were embedded in pH-neutralized nerve pre-hydrogel solution and then added to PDMS microwells. Cultures regained physiological morphology for 24 h before treatment with HPNE EVs/PANC-1 TEVs ± anti-IL8 neutralizing antibody (green pipette) for 48 h, at which time either CM was collected and SCs were imaged, or HPNE or PANC-1 cells were seeded atop the hydrogels (green). Invasion co-cultures were maintained with continual EV/TEV ± anti-IL8 treatment for 72 h. Alternatively, Transwell® invasion assays were performed with HPNE or PANC-1 using nerve dECM hydrogel and SC-CM ± anti-CCL2 (blue pipette). All invasion assays were imaged upon conclusion

Upon reaching 80% confluency, HPNE and PANC-1 cells (passage < 25) were incubated with FBS-free DMEM for 24 h. Conditioned media (CM) were collected, and cells were harvested. Harvested cells presented ≥ 90% viability as quantified by trypan blue (Thermo 15–250-061). CM were centrifuged at 1000 rpm for 5 min and aliquoted for molecular weight filtration (MWF), ExoQuick Ultra kit isolation (EQU), or ultracentrifugation (UC) (Fig. 1A). MWF samples were centrifuged in 100-kDa molecular weight cut-off centrifugal filters (MWCO; Sigma-Aldrich UFC910024) at 2000 rpm until concentrated tenfold; Western blot samples were rinsed twice with phosphate-buffered saline (PBS; VWR 97062–948). EQU-isolated EVs were isolated following ExoQuick Ultra (Amsbio EQULTRA-20TC-1) manufacturer’s instructions. UC samples were centrifuged using the Beckman Coulter ultracentrifuge (Brea, CA) at 200,000 × g at 4 °C for 90 min [19]; the pellet was resuspended in PBS and centrifuged for 3 h. These EVs were subject to size distribution and concentration analyses via NanoSight as described below.

For Luminex analysis as described below, EVs were lysed 5X RIPA buffer (250 mM Trizma-HCl (Sigma-Aldrich T3253), 750 mM NaCl (VWR BDH9286), 5% NP-40 (Sigma-Aldrich NP40S), 2.5% (w/v) sodium deoxycholate (SD; Sigma-Aldrich D6750), 0.5% (w/v) sodium dodecyl sulfate (SDS; Sigma-Aldrich 75,746)) to a final concentration of 4X + 1% Halt protease/phosphatase inhibitor cocktail (Thermo 78,440). The samples were agitated at 4 °C for 30 min and centrifuged at 12,700 rpm for 10 min.

### NanoSight nanoparticle tracking analysis

EV samples were diluted and added to NanoSight NS300 (Malvern Panalytical, UK). Sample readings (technical replicates *n* = 5) were conducted for 30–60 s per read with continuous syringe flow (*SI 1–6*). Sample concentration and size distribution results were adjusted according to the dilution factor.

### EV proteomic analysis

PANC-1 and HPNE EVs were isolated as described above (biological replicates *n* = 5). Protein samples were reduced, alkylated, and digested using filter-aided sample preparation with sequencing grade-modified porcine trypsin (Promega) [20]. Tryptic peptides were then separated by reverse phase XSelect CSH C18 2.5 µm resin (Waters) on an in-line 150 mm × 0.075 mm column using an UltiMate 3000 RSLCnano system (Thermo). Peptides were eluted using a 60-min gradient from 98:2 to 65:35 buffer A:B ratio (i.e., buffer A containing 0.1% formic acid and 0.5% acetonitrile, buffer B containing 0.1% formic acid and 99.9% acetonitrile). Eluted peptides were ionized by electrospray (2.2 kV) followed by mass spectrometric analysis on an Orbitrap Exploris 480 mass spectrometer (Thermo). To assemble a chromatogram library, six gas-phase fractions were acquired on the Orbitrap Exploris with 4 m/z DIA spectra (4 m/z precursor isolation windows at 30,000 resolution, normalized AGC target 100%, maximum inject time 66 ms) using a staggered window pattern from narrow mass ranges using optimized window placements. Precursor spectra were acquired after each DIA duty cycle, spanning the m/z range of the gas-phase fraction (i.e., 496–602 m/z, 60,000 resolution, normalized AGC target 100%, maximum injection time 50 ms). For wide-window acquisitions, the Orbitrap Exploris was configured to acquire a precursor scan (385–1015 m/z, 60,000 resolution, normalized AGC target 100%, maximum injection time 50 ms) followed by 50 × 12 m/z DIA spectra (12 m/z precursor isolation windows at 15,000 resolution, normalized AGC target 100%, maximum injection time 33 ms) using a staggered window pattern with optimized window placements. Precursor spectra were acquired after each DIA duty cycle.

### Bioinformatic analysis

Proteomic data were searched using an empirically corrected library, and a quantitative analysis was performed to obtain a comprehensive proteomic profile. Proteins were identified and quantified using EncyclopeDIA and visualized with ScaffoldDIA using 1% false discovery thresholds at both the protein and peptide levels [21]. Protein-exclusive intensity values were assessed for quality using ProteiNorm [22]. The data were normalized using VSN, and statistical analysis was performed using linear models for microarray data (limma) with empirical Bayes (eBayes) smoothing to the standard errors [23, 24]. Proteins with an FDR-adjusted *p*-value < 0.05 and a fold change > 2 were considered significant. Significant protein results were imported into QIAGEN IPA to distinguish relevant pathways. Proteins associated with IL-8 signaling were mapped using ScaffoldDIA and VolcanNoseR.

### dECM nerve hydrogel

Porcine sciatic nerves (Tissue Source LLC, Zionsville, IN) were decellularized as previously described to remove cells while retaining ECM components (Fig. 1B) [18]. Briefly, porcine sciatic nerves stored frozen at − 80 °C until decellularization, which began with thawing at 37 °C. All steps were performed with agitation at room temperature unless otherwise stated. Nerve epineuria were removed, and nerves were cut into 1-inch sections. The nerves were then rinsed in ddH_2_O for 7 h. Samples were subjected to the following washes: 125 mM SB-10 (Sigma-Aldrich D4266) in 50 mM Na/10 mM phosphate buffer (Buffer 1; NaCl (VWR BDH9286), sodium dihydrogen phosphate monohydrate (VWR BDH9298), and disodium hydrogen phosphate heptahydrate (VWR BDH9296); 18 h)); 100 mM Na/50 mM phosphate buffer (Buffer 2; 15 min); 3% (w/v) SD/0.6 mM SB-16 (Sigma-Aldrich H6883) in Buffer 1 (2 h); Buffer 2 (15 min × 3); SB-10 (7 h); Buffer 2 (15 min); SD/SB-16 (1.5 h); and Buffer 1 (15 min × 3). A 0.773% (w/v) MgCl_2_ (VWR BDH9244) solution was created with Buffer 1, DNase (Sigma-Aldrich D4527) was diluted at 0.446% (w/v) in 0.15 mM NaCl, and a 75 U/ml DNase solution was made by combining the two resulting solutions. Nerves were incubated in DNase for 3 h without agitation and then rinsed in Buffer 1 (1 h × 3). Chondroitinase ABC (Sigma-Aldrich C3667) was diluted at 0.2 U/ml PBS, and nerves were incubated with chondroitinase ABC for 16 h without agitation. Finally, nerves were rinsed with PBS for 3 h, and the samples were stored dry at − 20 °C. Decellularized nerves were lyophilized for 3 days, chopped finely, and digested in 1 mg/ml pepsin (Sigma-Aldrich P7000)-HCl solution (Sigma-Aldrich 320,331) at 12 mg tissue/ml for 3 days at 400–500 rpm, creating nerve dECM pre-hydrogel solution which was subsequently used for cell culture.

### Platform fabrication

Silicon wafers were patterned with cylindrical columns 200 µm tall and 4 mm in diameter using photolithography and were utilized to fabricate PDMS microwells [18]. The microwells were plasma-cleaned and treated with 1% poly(ethyleneimine) (Sigma-Aldrich 181,978; 10 min) and 0.1% glutaraldehyde (Sigma-Aldrich G6257; 30 min) before culture setup as described below.

### EV inhibition

Nerve pre-hydrogel was combined with 10% M199 (Sigma-Aldrich M0650) and pH neutralized with 1 M NaOH (Sigma-Aldrich 415,413). sNF96.2 cells (passage < 10) were resuspended in medium according to Eq. 1 and added to the pH neutralized hydrogel at 1.5 million/ml. The mixture was added to PDMS microwells, topped with plasma-cleaned PDMS lids, and incubated at 37 °C for 30 min. Complete medium was added to the cultures, PDMS lids were removed, and cultures were maintained at 37 °C for 1 day to regain morphology (Fig. 1C). EVs (50 mg/ml) ± anti-IL-8 neutralizing antibody (2 µg/ml; Novus Biologicals MAB208) were added to each culture on day 2. Cultures were maintained for 2 additional days with fresh media/treatments added every 24 h.

1 $$\text{Cell medium volume}=\left(\text{Hydrogel volume }\times 0.1\right)-\text{NaOH volume}$$

### Invasion assays

A day before passaging, SCs were incubated with cell tracking live dye in medium (1/20; Abcam ab269446) for 24 h at 37 °C. Nerve pre-hydrogel solutions were pH neutralized, and SCs were embedded. Cultures were maintained for 1 day and then treated per EV inhibition for 2 days. HPNE or PANC-1 cells (5 K cells/well) were then seeded atop each hydrogel, and complete SC media ± EV (HPNE) or TEV (PANC-1) ± anti-IL-8 were added. Co-cultures were maintained for 3 days with fresh media/treatments replenished every 24 h (biological replicates *n* ≥ 3, technical replicates *n* = 3).

Additionally, Transwell® invasion assays were performed. Briefly, 30 µl of nerve pre-hydrogel was pH neutralized, added to the upper chamber of 6.5 mm, 8.0 µm pore Transwell® inserts (Sigma-Aldrich CLS3464), and allowed to polymerize at 37 °C. PANC-1 or HPNE cells were added on top of the hydrogel (50 K cells/insert). Cells were allowed acclimate for 24 h before CM collected from EV inhibition studies were added to the lower chamber (SC-EV CM for HPNE cells, SC-TEV CM for PANC-1 cells) ± anti-CCL2 (4 mg/ml; R&D MAB679). Cultures were then maintained for an additional 24 h (biological replicates *n* ≥ 4, technical replicates *n* = 3).

### Immunofluorescence and confocal microscopy

Microwell cultures were incubated in 3.7% formaldehyde at 4 °C for 1 h. For Transwell® cultures, hydrogels were first discarded, and the upper insert chamber was swabbed to remove non-invaded cells prior to fixing. Fixed samples were rinsed at room temperature with PBS, permeabilized with 0.05% Triton X-100 in PBS (5 min) and blocked with 1% bovine serum albumin (BSA) in PBS (30 min-1 h). Rabbit anti-GFAP (1/1000; Dako Z033429-2) in 1% BSA was added to each culture and incubated at 4 °C overnight. Samples were rinsed at room temperature with 0.05% Tween-20 in PBS (5 min × 3) and incubated with goat anti-rabbit (1/500; Thermo A11011), phalloidin 488 (1/400; Thermo A12379), and/or DAPI (1/2500; Thermo D1306) in 1% BSA away from light (1 h). Biological replicates *n* ≥ 3 and technical replicates *n* = 3. All samples were stored in fresh PBS at 4 °C and imaged with the Olympus IX-83 confocal microscope (× 20 magnification, 1–2 × zoom, 2–5 µm steps).

### Luminex multiplex analyses

Lysed EVs (biological replicates *n* ≥ 1, technical replicates *n* ≥ 4) were concentrated with 3-kDa (Fisher UFC800396) or 10-kDa MWCO filters (Sigma UFC201024) and characterized via Luminex Assay for CD9, CD63, CD81, syntenin-1, integrin α4β1, and cytochrome C (Thermo Fisher) or IL-8 (R&D Systems) according to the manufacturer’s instructions. Results were normalized by total EV protein content (BCA). SC-laden hydrogels were incubated in FBS-free DMEM overnight on day 3 of culture (biological replicates *n* = 4, pooled). Conditioned media were concentrated with 3-kDa MWCO filters and analyzed for GDNF, IL-1β, IL-6, IL-8, MMP-2, and CCL2 according to the manufacturer’s instructions (technical replicates *n* = 3). SC culture DNA was quantified (Qiagen 69,506, Promega E2670) and used to normalize results by relative cell number.

### Image analysis

All image analysis was conducted blindly. GFAP immunofluorescence images were imported into Fiji, and Z-stacks were compressed to maximum intensity. Images were auto-thresholded, and RGB Measure Plus-determined intensities were normalized by nuclei count. Debris was removed from the background of figure images using a custom MATLAB code. For co-culture invasion studies, confocal reflectance determined the top of each hydrogel in which at least 50% of the hydrogel spanned across the image. Nuclei of invading cells per Z-slice were manually tracked with MTrackJ, and average and maximum invasion depths alongside percentages of invaded cells per slice were tallied. Transwell® images were compressed using maximum intensity and imported into MATLAB, where nuclei co-localized with actin staining were counted.

### Statistical analysis

T-tests and two-way ANOVAs with Tukey’s post-hoc tests as specified in corresponding figure captions were performed using GraphPad Prism 9.4. Significant outliers were identified and removed using GraphPad Prism Outlier Calculator (*α* = 0.05).

## Results

### The isolation of EVs using MWF is confirmed

**Fig. 2:**
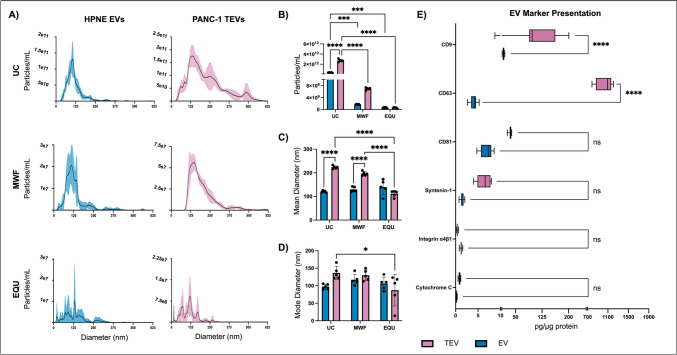
EV isolation and characterization. **A** NanoSight nanoparticle tracking analysis plotted the size distribution of HPNE (healthy) and PANC-1 (PDAC) EV samples. **B** Total particle concentration along with **C**, **D** mean and mode particle diameters in EV samples as collected by NanoSight was compared. Technical replicates *n* = 5. Two-way ANOVAs with Tukey’s post-hoc tests, UC/MWF/EQU and EV/TEV. **E** EV markers CD9, CD63, CD81, syntenin-1, integrin α4β1, and cytochrome C were quantified with Luminex multiplex analysis. Two-way ANOVA with Šidák’s post-hoc test, Luminex analytes, and EV/TEV. Technical replicates *n* ≥ 6. ns, not significant, *p < 0.05, ***p < 0.005, ****p < 0.0001

PDAC TEVs and healthy cell-derived EVs were collected from CM of PANC-1 and HPNE cells, respectively. EVs and TEVs were isolated via UC, MWF, and EQU and were assessed for particle size distribution using NanoSight nanoparticle tracking analysis tool (Fig. 2A). This revealed EQU-isolated TEVs to have significantly different mean (*p* < 0.0001) and mode diameters versus UC-isolated samples (*p* < 0.05), whereas MWF-isolated samples were similar in size to UC samples (Fig. 2B and C). UC- and MWF-isolated TEVs were also significantly larger on average than EVs isolated in the same manner (*p* < 0.0001). While MWF and EQU collected significantly fewer particles than UC (Fig. 2D), MWF is a more practical alternative to UC in terms of time and equipment accessibility. Given the similarity in isolated particle sizes to UC, MWF was chosen for subsequent EV isolations.

Luminex multiplex analysis was performed to confirm the presence of EV markers CD9, CD63, CD81, syntenin-1, and integrin α4β1 in MWF samples (Fig. 2E**)**. Both EVs and TEVs were found to be positive for all markers, although TEVs presented significantly higher CD9 and CD63 levels than EVs (*p* < 0.0001). CD81 and syntenin-1 levels were also slightly elevated in TEVs but not significantly. Interestingly, EVs had slightly, though not significantly, higher levels in integrin α4β1 than TEVs. Mitochondrial and apoptotic body marker cytochrome C was found in negligible quantities in either sample sets.

### IL-8 signaling-associated proteins are significantly elevated in pancreatic TEVs

**Fig. 3:**
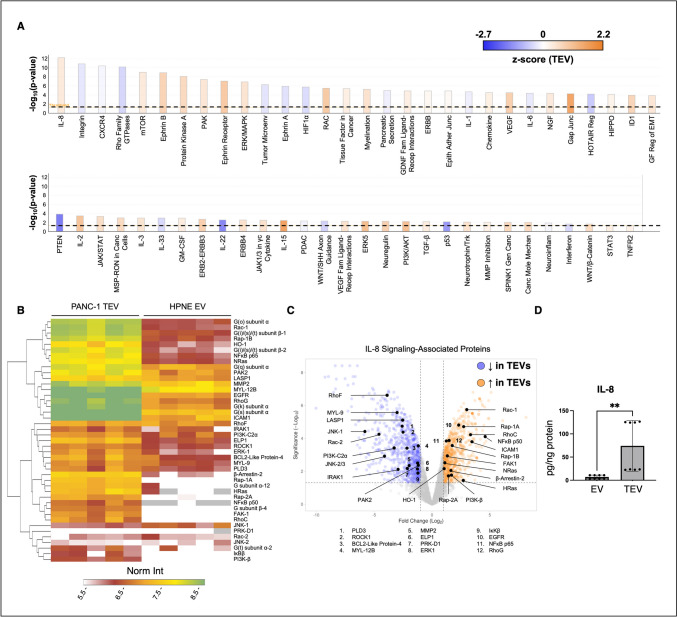
EV protein and pathway analysis. **A** Differentially expressed proteins from proteomics were imported into Qiagen IPA to determine significant pathways up- and downregulated in PANC-1 TEVs. Biological replicates *n* = 5. **B**, **C** Significantly altered proteins related to IL-8 signaling were highlighted in the heatmap and volcano plot. Biological replicates *n* = 5. Significance (− log_10_) ≥ 1.3, and fold change (log_2_) ≥|1| are significant. **D** The presence of IL-8 in EV samples, normalized by total protein per BCA assay, was confirmed via Luminex assay. Biological replicates *n* = 2, technical replicates *n* = 4. Unpaired t-test. **p < 0.01

Proteomics examined EV and TEV cargoes with the potential to activate SCs. The results highlighted 1550 differentially expressed proteins, with 815 proteins decreased and 735 proteins increased in TEVs. These proteins were then subjected to pathway analysis to identify potential contributors to PNI (Fig. 3A). Here, IL-8 signaling received among the highest significance scores, i.e., − log10(*p*-value) of 12.2, with a *z*-score of 0.667, indicating an elevation in TEVs. Proteins involved in IL-8 signaling were then visualized via heatmap and volcano plot (Fig. 3B and C). PI3K-beta and NFκB subunits p50 and p65, both downstream products of IL-8 uptake, were shown to be significantly higher in TEVs. Furthermore, NFκB p50 but not p65 nuclear translocation was found to be significantly increased in SCs treated with TEVs (*SI 7*). In probing IL-8 content, EVs were found to contain 7.1 ± 4.0 pg IL-8/ng of total protein, while TEVs showed levels of 74.1 ± 54.7 pg/ng protein (Fig. 3D).

## IL-8 increases SC activation and PNI-associated protein secretion

**Fig. 4:**
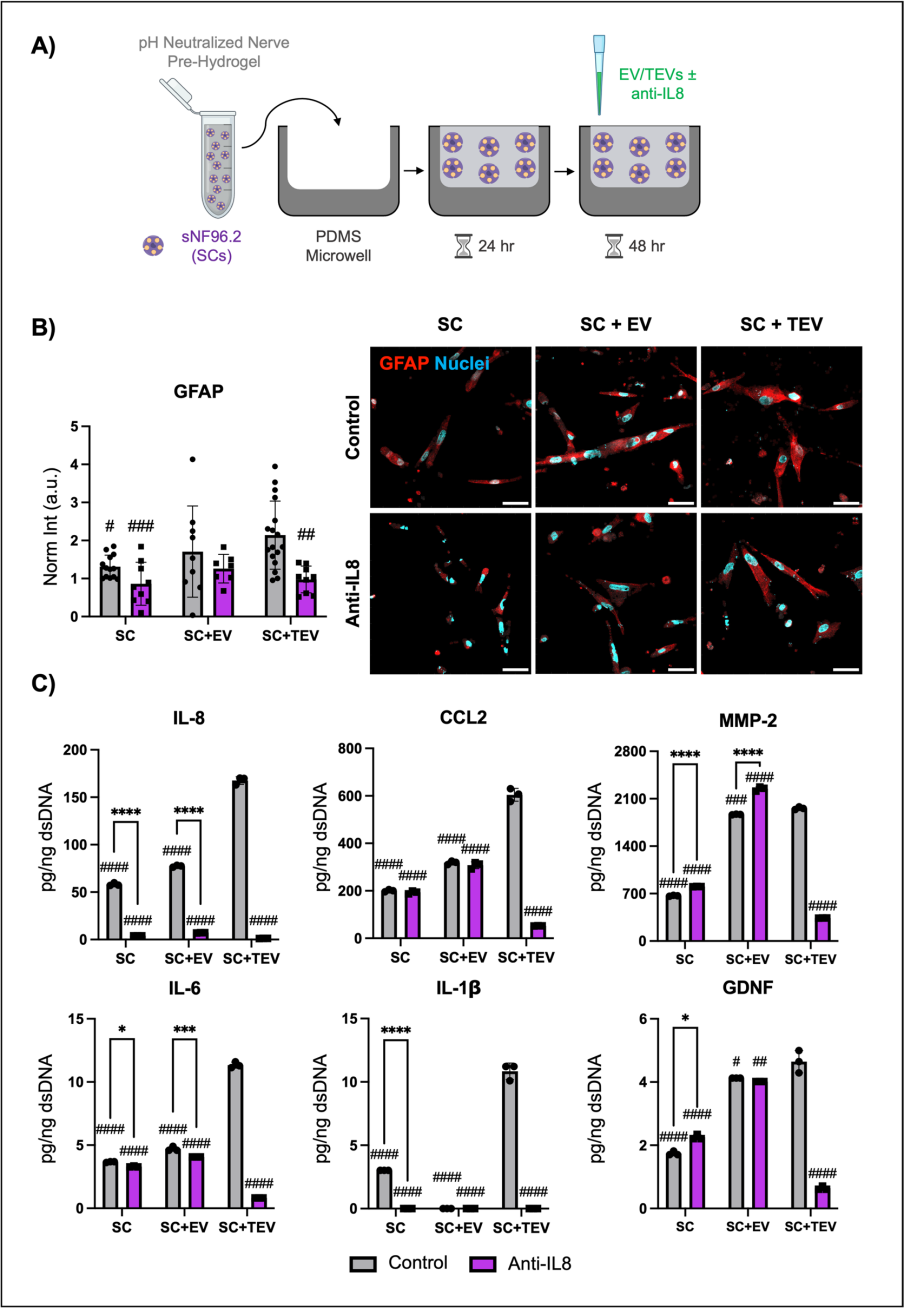
Phenotypic and secretome changes via IL-8. **A** SCs (purple) were embedded in nerve dECM pre-hydrogel (pink) and then added to PDMS microwells to polymerize. After 1 day of culture, SCs were treated with EV/TEVs ± anti-IL8 once every 24 h for 2 days. **B** Immunofluorescence staining of GFAP was performed to determine SC activation. Scale: 50 µm. Biological replicates *n* ≥ 3, technical replicates *n* = 3. **C** Luminex results assessed IL-8, CCL2, MMP-2, IL-6, IL-1β, and GDNF produced by SCs, which were normalized by dsDNA to demonstrate secretion per cell. Biological replicates *n* = 4 (pooled), technical replicates *n* = 3. Two-way ANOVAs with Tukey’s post-hoc tests, control/anti-IL8 and SC/SC + EV/SC + TEV. *p < 0.05, **p < 0.01, ***p < 0.005, ****p < 0.0001. # indicates significant difference from SC + TEV control samples

We then implemented our dECM nerve hydrogels previously optimized to observe neural cell responses to disease in a controlled and physiomimetic manner [18, 25]. Embedded SCs were treated with EVs or TEVs ± IL-8 neutralizing antibody to elucidate the effect of IL-8 on SC activation (Fig. 4A). To assess phenotypic evidence of SC activation, treated groups were evaluated for relative levels of SC activation marker GFAP (Fig. 4B). TEV-treated SCs showed significantly higher GFAP levels than untreated SCs. This increase in activation levels was then depleted following IL-8 inhibition, while EV-treated and control SCs showed no changes associated with IL-8. Although GFAP levels were higher in TEV-treated SCs than EV-treated SCs, the differences were not significant, requiring further evidence of activation.

Secretome analysis was then conducted, which showed that TEV-treated SCs secreted significantly higher levels of IL-8, CCL2, MMP-2, IL-6, IL-1β, and GDNF (Fig. 4C) over both control and EV-treated SCs. There was a significant increase in IL-8 production by EV- (77.3 ± 1.0 pg/ng dsDNA) and TEV-treated SCs (167.6 ± 4.1 pg/ng dsDNA) in comparison to untreated control samples (58.1 ± 1.6 pg/ng dsDNA), but TEV-treated SCs secreted significantly higher IL-8 levels than EV-treated SCs (*p* < 0.0001). Following IL-8 inhibition, control (3.9 ± 0.0 pg/ng DNA), EV-treated (7.2 ± 0.2 pg/ng dsDNA), and TEV-treated SCs (1.2 ± 0.01 pg/ng dsDNA) all decreased IL-8 secretion. These findings confirmed heightened IL-8 signaling in SCs can be attributed to TEVs and their cargo IL-8. Most notably, CCL2 (604.5 ± 27.3 pg/ng dsDNA) and MMP-2 concentrations (1959.3 ± 21.0 pg/ng dsDNA) were exponentially greater in TEV-treated SCs than any other secreted factors; however, neutralizing IL-8 increased MMP-2 secretion in control and EV-treated SCs.

### Pancreatic TEV/IL-8-primed SCs enhance PDAC cell invasiveness via CCL2

**Fig. 5:**
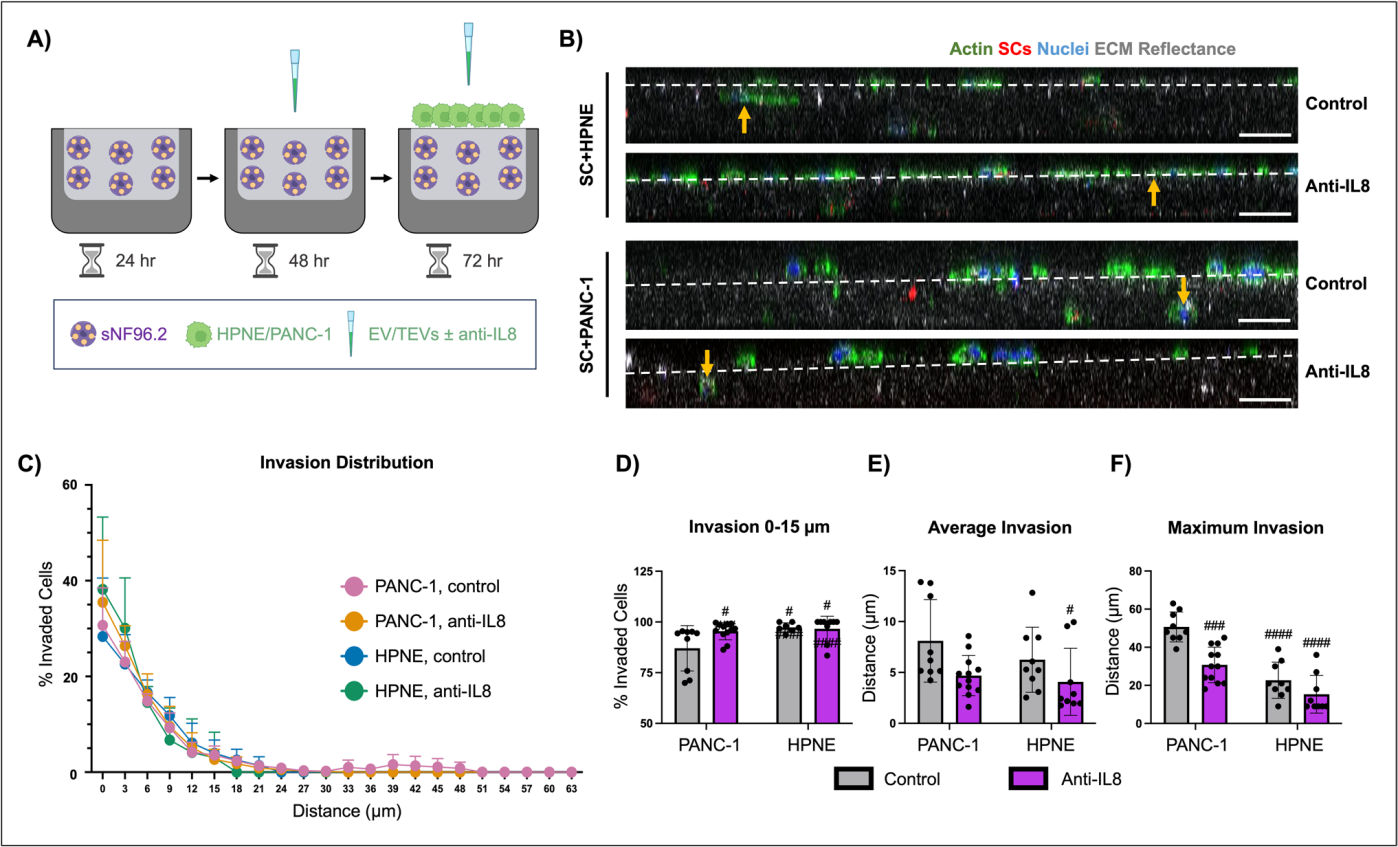
IL-8-induced PDAC invasion. **A** SCs were embedded in nerve dECM hydrogel and treated with EV/TEVs ± anti-IL8 as previously described. On day 3, HPNE or PANC-1 cells were seeded atop SC-laden hydrogels and EV/TEV ± anti-IL8 treatment continued for 3 additional days. Biological replicates *n* ≥ 3, technical replicates *n* = 3. **B**–**F** Immunofluorescence was performed and analyzed manually by counting epithelial cells per *Z*-slice/3 µm and normalizing to the total invaded cells. Scale: 50 µm. **D** The percentage of cells present in the top 15 µm were plotted. **E**, **F** Average and maximum invasion depths were compared. Two-way ANOVAs with Tukey’s post-hoc tests (**D**–**F**), control/anti-IL8 and PANC-1/HPNE. *p < 0.05, ***p < 0.005, ****p < 0.0001. # indicates significant difference from SC + TEV control samples

To model PNI in vitro, dECM nerve hydrogels were embedded with live dye-tagged SCs and primed with EVs or TEVs ± anti-IL8 (Fig. 5A). The appropriate epithelial cells (HPNE for EV-primed cells or PANC-1 for TEV-treated cells) were then seeded atop SC-laden hydrogels, and co-culture was maintained with continuous EV or TEV ± anti-IL8 treatment. Culture concluded on day 6, after which immunofluorescence was performed and analyzed via confocal microscopy (Fig. 5B). Invaded epithelial cells per Z-slice were counted; the total number of invaded cells were used to normalize the counts at each *Z*-slice, resulting in a percentage of total invaded cells at each depth (Fig. 5C and D). Cell count per depth was then used to calculate the average and maximum invasion depths (Fig. 5E and F).

Here, a higher percentage of invading HPNE and PANC-1 cells treated with anti-IL8 were located at invasion depths of 0 and 3 µm compared to control cells. In fact, a significantly lower percentage of invading control PANC-1 cells (87.0 ± 11.2%) was located in the top 15 µm of the hydrogel compared to PANC-1 treated with anti-IL8 (95.5 ± 4.3%) and either HPNE treatment groups. The average invasion depth travelled by control PANC-1 cells (8.1 ± 4.1 µm) was only slightly greater than control HPNE cells (6.3 ± 3.2 µm) and PANC-1 treated with anti-IL8 (4.7 ± 2.0 µm). Still, control PANC-1 samples displayed significantly greater maximum invasion depths (50.7 ± 7.9 µm) than PANC-1 treated with anti-IL8 (30.8 ± 9.3 µm) and control HPNE (22.7 ± 9.5 µm).

**Fig. 6:**
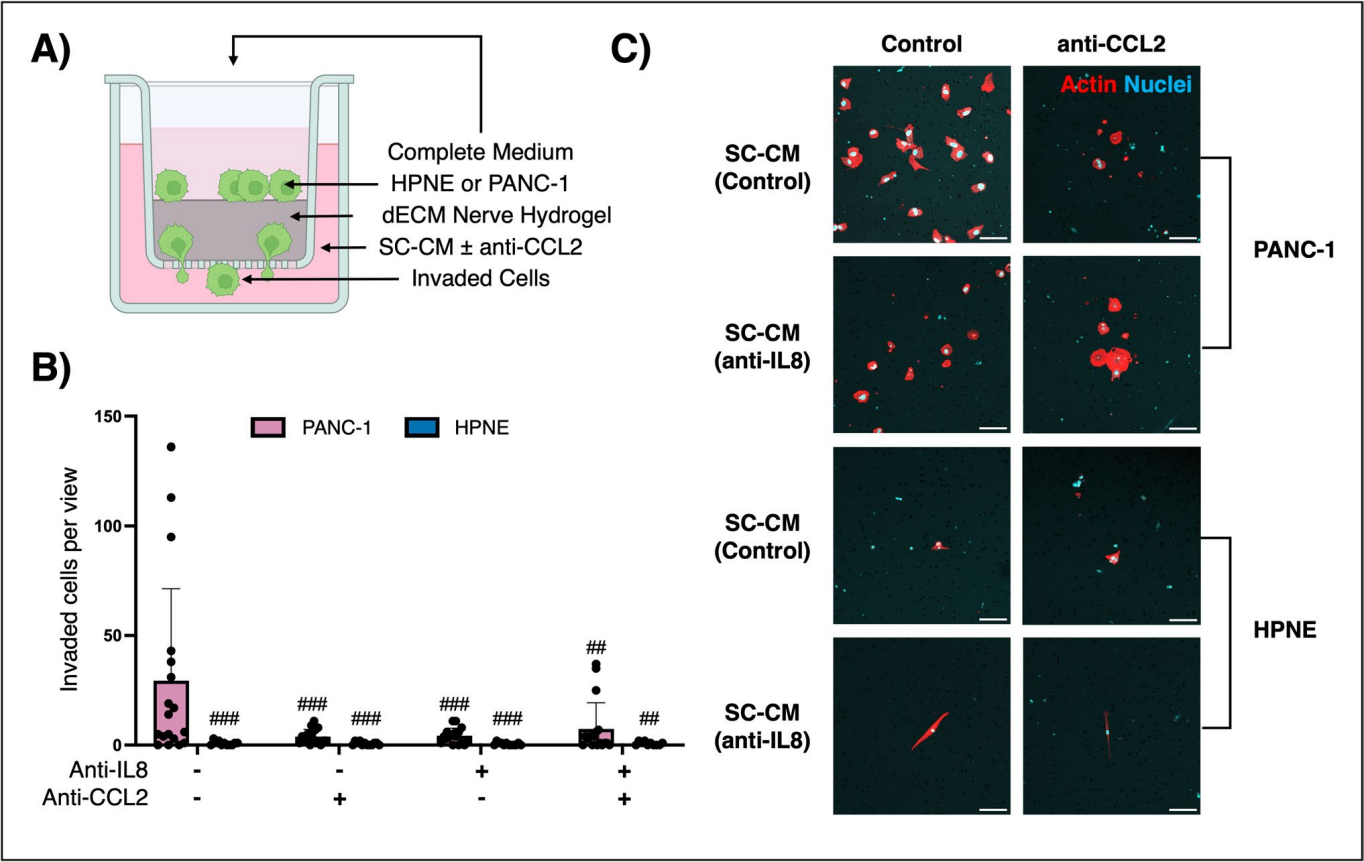
CCL2-induced PDAC invasion. **A** HPNE or PANC-1 cells (green) were seeded on nerve hydrogel in the upper Transwell® chamber with complete media (dark pink). SC-CM from cultures treated with EVs (HPNE) or TEVs (PANC-1) ± anti-IL8 was added to the lower chamber ± anti-CCL2 (light pink). Biological replicate *n* ≥ 4, technical replicates *n* = 3. **B**, **C** Immunofluorescence visualized the number of invaded cells. Scale, 100 µm. Two-way ANOVAs with Tukey’s post-hoc test, control/anti-IL8/anti-CCL2 and PANC-1/HPNE. **p < 0.01, ***p < 0.005, # indicates significant difference from SC + TEV control samples

To ascertain the factors secreted by SCs that propagate this invasion, Transwell® invasion assays were performed using CM collected from SCs treated with EV/TEV ± anti-IL8. Using Transwell® invasion assays for this application allows for the targeting of specific cytokines secreted by SCs without any confounding factors coming from HPNE or PANC-1 cells. Revisiting the secretome analysis, CCL2, a chemokine implicated in cervical cancer, prostate cancer, and PDAC PNI [26–28], was identified as a potential player in guiding PDAC cells towards SCs; therefore, CCL2 was inhibited in CM via neutralizing antibody. Transwell® inserts were coated with nerve hydrogel before PANC-1 or HPNE cells were seeded in the upper chamber (Fig. 6A). After seeding, CM ± anti-CCL2 was added to the lower chamber. Culture was maintained for 48 h before the hydrogels were discarded and the upper chamber was swabbed clean to leave only invaded cells.

While HPNE cells exhibited limited invasion regardless of treatment, the number of invading PANC-1 cells was significantly higher in the absence of inhibitors (29.4 ± 41.9 cells) than with anti-CCL2 (3.9 ± 3.2 cells), anti-IL-8 (4.3 ± 3.4 cells), and combined treatment groups (7.4 ± 11.9 cells) (Fig. 6B and C). Moreover, control CM-treated PANC-1 invaded at significantly higher numbers than control CM-treated HPNE cells (0.8 ± 1.0 cells). While anti-CCL2 effects in limiting PANC-1 invasion were slightly more effective than anti-IL8, there was no significant benefit of inhibiting one chemokine over another nor in neutralizing both.

## Discussion

In this study, we utilized in vitro dECM nerve hydrogel testbed to study SC response to PDAC TEVs and healthy cell EVs in relation to PNI. While major tissue engineering techniques were first published in the late 1990s, the process of decellularization is relatively new, with numerous protocols propagating in literature within the last 20 years [29]. Decellularization is a complex process in which tissues are chemically, enzymatically, or mechanically altered to rid them of native cells while retaining extracellular matrix composition and structure [17]. Early work focused on the regenerative medicine potential of these tissues, but biomedical engineers have garnered a newfound interest in utilizing decellularized tissues as scaffolds to investigate cancer mechanisms and therapeutics in vitro [30]. Within the last 5 years, decellularized PDAC patient samples, xenografts, and/or healthy pancreases have been employed as therapeutic testbeds designed to observe PDAC drug response in a biomimetic microenvironment [31–34]. Still, few dECM scaffolds have been applied to investigate the mechanisms behind cancer-nerve crosstalk. Presently, in vivo studies are a standard route for examining PNI; however, animal models often are expensive, difficult to manage, and translate poorly to the clinic [12, 13]. Three-dimensional in vitro studies of PNI and tumor innervation also overwhelmingly utilize tumor-derived commercial biomaterials, which could influence Schwann cell behavior independent of TEV treatments and potentially leaving a gap in our understanding of the phenomena [16, 35–38].

Since discovering TEV involvement in cancer, researchers have mainly focused on exosomes, but microvesicles have recently been embraced as a player in cancer progression. In the present study, NanoSight analysis found PANC-1 to release larger TEVs on average compared HPNE EVs. Santana et al. compared healthy and tumor EVs from breast, brain, and pancreatic cells and presented similar size distributions to the present study [39]. Despite this, Luminex® results proved PANC-1 TEVs to have slightly higher syntenin-1 levels, a protein involved in exosome biosynthesis, than HPNE EVs. Additionally, TEVs presented with significantly elevated CD9 and CD63 concentrations, along with a moderate elevation in CD81 levels. These results could be partially attributed to the overproduction of TEVs versus EVs; however, CD9 and CD63 expression is associated tumorigenicity in PDAC [40]. Syntenin-1 also contributes to PDAC cell proliferation and invasion [41]. CD81 is known to be expressed in PDAC cell and patient EVs, but its contribution to the disease is less apparent [42, 43]. Similarly, while integrin α4β1 is slightly decreased in TEVs, its role in PDAC exosomal signaling is relatively unexplored.

Our results suggest the role of IL-8 and CCL2 in Schwann cell activation, and PNI. IL-8 is a prognostic factor in many cancers, including PDAC [44]. In fact, PDAC patient serum contains significantly higher IL-8 concentrations than healthy patients and patients of other gastrointestinal cancers [45, 46]. IL-8 promotes a stem-like phenotype, proliferation, and chemoresistance in cancer cells as well as angiogenesis [44, 47, 48]. Preclinical and clinical studies have found cancer-associated fibroblast-derived IL-8 to increase PDAC invasiveness, while *Fusobacterium nucleatum* in the tumor microenvironment promotes IL-8 secretion and autocrine signaling in PDAC cells to boost invasion [49–51]. Notably, colorectal cancer cells induce IL-8 production in SCs, and inhibiting IL-8 decreased SC activation [52]. Our study suggests IL-8 plays a similar role in PDAC PNI; more specifically, PDAC TEVs traffic IL-8 to SCs to promote activation and PNI in vitro. IL-8 increased GFAP expression by SCs, which has also been highlighted in as a sign of activation in nerve injury and thyroid, head and neck, skin, colon, and pancreatic cancers [53, 54]. TEV/IL-8-activated SCs also upregulated their secretion of GDNF, IL-6, IL-1β, MMP-2, and CCL2. Cavel et al. demonstrated that macrophage GDNF secretion facilitates PDAC PNI by promoting downstream Erk signaling [55]. IL-6 and IL-1β are also documented in supporting SC activation in PNI and other neurodegenerative diseases [27, 56–60]. MMP-2 contributes to cancer-nerve crosstalk in breast, prostate, and pancreatic cancers, whereas neural cell secretion of CCL2 is a contributor to cervical, prostate, and pancreatic cancer PNI [26–28, 61–63]. It should also be noted that SCs form bridges to facilitate axonal regeneration post-nerve injury [64], a phenomenon which was also recently shown to facilitate PDAC PNI [4]. Future work may assess these behavioral and/or morphological changes of SCs in our nerve-mimetic models.

From this, we can conclude that PANC-1 TEV-driven PNI may be limited by targeting either IL-8 or CCL2 in vitro, but there may be no additional benefit with combinational treatment. Validating these results through selective receptor inhibitions and animal models of PNI may further elucidate the viability of these therapeutic strategies to curb PDAC PNI. Targeted therapies for PDAC PNI have gained more attention since its prominence has become apparent in recent years, with current literature highlighting pharmacological and genetic engineering approaches to limit PNI in vitro and in vivo [3, 4, 65]. The findings presented here highlight a promising route for the treatment of PDAC PNI using monoclonal antibodies.

## Limitations of this study

The priority of the present study was to determine how TEVs as a whole impact PDAC PNI; therefore, all EVs under 1 µm in diameter were examined. Within that range, we found PANC-1 TEVs to be comprised of larger EV subpopulations than did HPNE EVs. Future work will explore the role of EV subtypes, e.g., exosomes versus microvesicles, in promoting PDAC invasiveness. Additionally, large oncosomes (diameters 1–10 µm), commonly produced by hyper-aggressive amoeboid cancer cells, have gained attention for their potential role in cancer [66, 67]. Continued investigations are needed to probe these larger EVs and how they influence PNI.

To confirm TEV-derived IL-8 and SC-secreted CCL2 promote PDAC PNI in vitro, function-blocking monoclonal antibodies were implemented to target each. We recognize that this shift in invasiveness could be attributed to SC-released IL-8 and CCL2 from TEV treatment, so subsequent studies will utilize CRISPR-Cas9 to knockout IL-8 in epithelial cells and/or CCL2 in SCs. Alternatively, CCL2 receptor CCR2 may be explored as a potential target for limiting IL-8/CCL2 signaling in PDAC PNI. Performing knockout of these proteins or receptors could complement the Transwell® assays in future work.

Finally, this study included one malignant and one benign epithelial cell line to validate the relationship between TEVs and PNI. Future work will incorporate a larger variety of PDAC cell lines along with patient-derived primary cells to further confirm this mechanism; moreover, while the FDA Modernization Act 2.0 has signaled a shift towards in vitro-based pre-clinical investigations, in vivo studies may be conducted in the future to corroborate the efficacy of targeting TEVs to limit PDAC PNI.

## Conclusions

Here, we conclude that TEVs prompt SC activation and CCL2 secretion via IL-8 cargoes. HPNE healthy epithelial and PANC-1 PDAC cells cultured within a previously optimized dECM nerve hydrogel was utilized as a disease modeling tool to establish a possible mechanism behind this interaction. Using our in vitro testbed, this IL-8/CCL2 cascade driven by TEVs magnified PANC-1 cell invasiveness towards SCs in vitro, which was limited through function-blocking monoclonal antibodies. Future pre-clinical studies with enhanced biodiversity will further solidify the importance of this mechanism and its potential as a therapeutic target for PDAC.

## Supplementary Information

Below is the link to the electronic supplementary material.

Supplementary file1 (MP4 13086 KB)

Supplementary file2 (MP4 4995 KB)

Supplementary file3 (MP4 10305 KB)

Supplementary file4 (MP4 710 KB)

Supplementary file5 (MP4 102 KB)

Supplementary file6 (MP4 112 KB)

Supplementary file7 (PNG 21635 KB)

## Data Availability

Data will be made available upon reasonable request to the corresponding author.
