# Supplementary figures and images for: Pancreatic cancer extracellular vesicles stimulate Schwann cell activation and perineural invasion in vitro via IL-8/CCL2
Source: In Vitro Model. 2025 Mar 7;4(1):45–58. doi: 10.1007/s44164-025-00083-w (PMC11950487; doi:10.1007/s44164-025-00083-w)

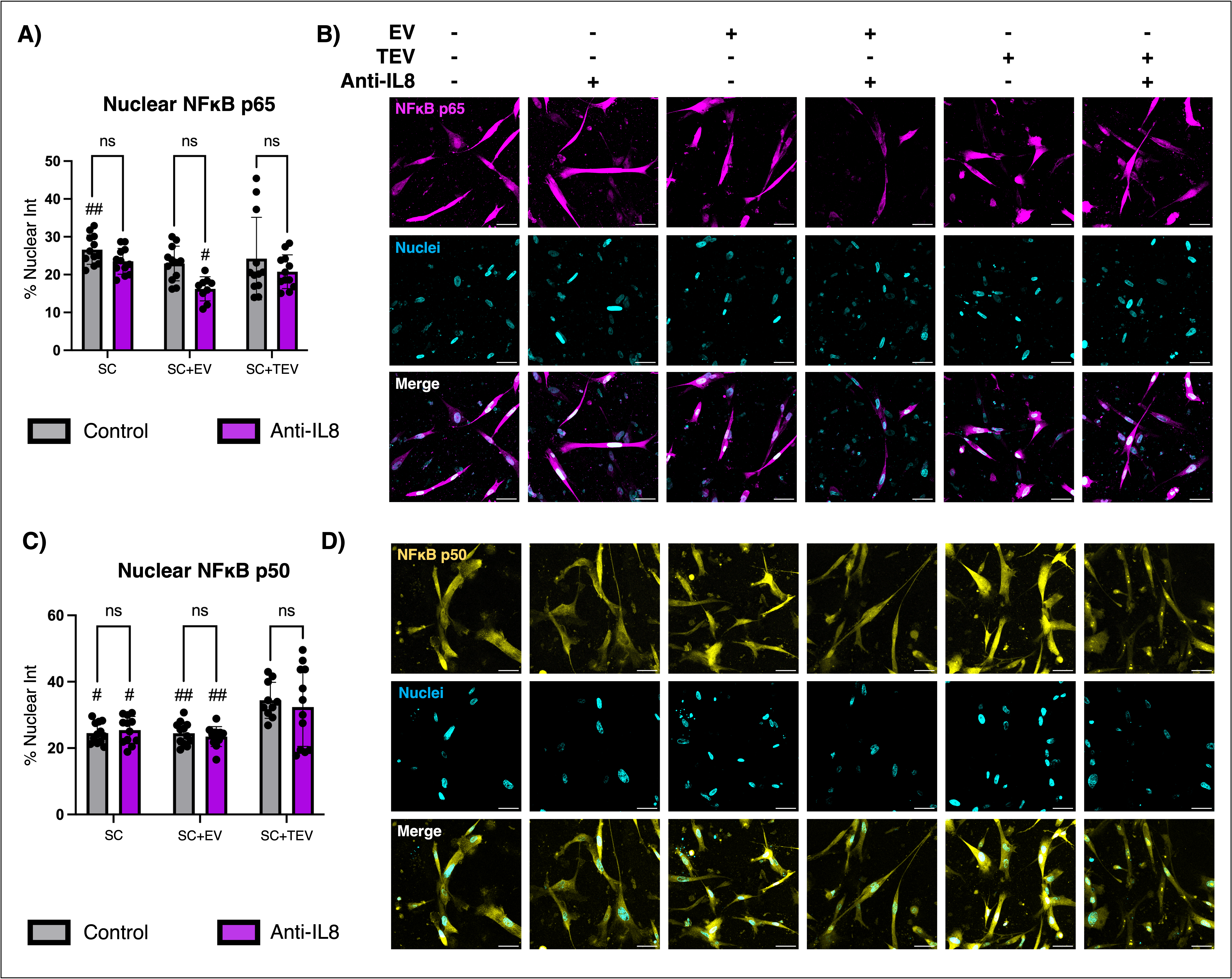

Supplement: Supplementary file 7 — Supplementary file7 (PNG 21635 KB) [file 44164_2025_83_MOESM7_ESM.png]
